# The Role of Natural and Semi-Synthetic Compounds in Ovarian Cancer: Updates on Mechanisms of Action, Current Trends and Perspectives

**DOI:** 10.3390/molecules28052070

**Published:** 2023-02-22

**Authors:** Md. Rezaul Islam, Md. Mominur Rahman, Puja Sutro Dhar, Feana Tasmim Nowrin, Nasrin Sultana, Muniya Akter, Abdur Rauf, Anees Ahmed Khalil, Alessandra Gianoncelli, Giovanni Ribaudo

**Affiliations:** 1 Department of Pharmacy, Faculty of Allied Health Sciences, Daffodil International University, Dhaka 1207, Bangladesh; 2 Department of Chemistry, University of Swabi, Anbar 23430, Pakistan; 3 University Institute of Diet and Nutritional Sciences, Faculty of Allied Health Sciences, The University of Lahore, Lahore 54000, Pakistan; 4 Dipartimento di Medicina Molecolare e Traslazionale, Università degli Studi di Brescia, Viale Europa 11, 25123 Brescia, Italy

**Keywords:** ovarian cancer, natural compounds, semi-synthetic compounds, medicinal chemistry, anti-metastasis, apoptosis

## Abstract

Ovarian cancer represents a major health concern for the female population: there is no obvious cause, it is frequently misdiagnosed, and it is characterized by a poor prognosis. Additionally, patients are inclined to recurrences because of metastasis and poor treatment tolerance. Combining innovative therapeutic techniques with established approaches can aid in improving treatment outcomes. Because of their multi-target actions, long application history, and widespread availability, natural compounds have particular advantages in this connection. Thus, effective therapeutic alternatives with improved patient tolerance hopefully can be identified within the world of natural and nature-derived products. Moreover, natural compounds are generally perceived to have more limited adverse effects on healthy cells or tissues, suggesting their potential role as valid treatment alternatives. In general, the anticancer mechanisms of such molecules are connected to the reduction of cell proliferation and metastasis, autophagy stimulation and improved response to chemotherapeutics. This review aims at discussing the mechanistic insights and possible targets of natural compounds against ovarian cancer, from the perspective of medicinal chemists. In addition, an overview of the pharmacology of natural products studied to date for their potential application towards ovarian cancer models is presented. The chemical aspects as well as available bioactivity data are discussed and commented on, with particular attention to the underlying molecular mechanism(s).

## 1. Introduction

Among gynecologic cancers, ovarian cancer is categorized as the third most prevalent cancer after cervical and uterine cancer, having high mortality rates in female subjects [1]. Epithelial ovarian malignancies, which include serous, mucinous, endometrioid, metastatic and clear cell carcinoma, represent the majority of ovarian diseases [2]. Intracavitary implantation, hematic and lymphatic pathways can all be exploited to transmit epithelial ovarian cancer, and the main route of spread is intraperitoneal metastases [3,4]. In the initial phases of the disease, patients are commonly asymptomatic, and 70% of patients are diagnosed at a later stage [5].

Nowadays, cytoreductive surgeries followed by platinum/paclitaxel-based chemotherapy are being considered as first-line approaches. Patients who receive this treatment, on the other hand, are prone to develop chemotherapeutic resistance and cumulative adverse effects, including nephrotoxicity [6]. To date, treatment of ovarian cancer is considered among the most challenging tasks in the field of oncology owing to its low survival rate (5 years < 40%) [7]. Consequently, novel drugs and alternative therapeutics for treatment or prevention of progression of ovarian cancer are needed.

Due to the adverse side effects associated with standard anticancer treatment, plant-derived products, alone and/or in combination with conventional anticancer agents, are being explored nowadays as adjuvant treatment to minimize adverse side effects [8].

Recently, several ovarian cancer prevention and early detection strategies have not shown expected or satisfactory results, which is partially attributable to the disease’s heterogeneity [9]. Increased DNA lesion repair, aberrant intracellular signal transmission, and drug metabolic inactivation are all promoted by genetic changes. Complete remission has instead been achieved by combining surgical intervention with genetic analysis [10]. In this context, the PARP inhibitor olaparib is administered to patients with *BRCA1* or *BRCA2* mutations [11]. It is also common practice to treat patients who have experienced a relapse after receiving platinum-based chemotherapy. Due to rapid tumor development and chemotherapy resistance, the period between treatments decreases progressively after a relapse.

As anticipated, to increase the survival quality of patients undergoing chemotherapeutic treatment, novel approaches are required. Botanical components are naturally occurring antioxidants or alkaloids with a long history in ethnopharmacology and characterized by the potential of being employed as therapeutic resources [12,13,14]. Natural products derived from plants are commonly thought of as nutritional supplements [15], meanwhile, plant-based constituents and products may be used as adjuvant therapies against ovarian cancer and for reduction of metastatic tumor size, and some examples have been reported in the literature.

With this review, we aim at highlighting the beneficial impacts of natural products, as single molecules and in combination, on ovarian cancer. Several scientific databases (Google Scholar, PubMed, Embase, etc.) were searched for records published from 2000 onwards by using the following keywords: ovarian cancer, etiology, risk of ovarian cancer incidence, natural products and ovarian cancer, ovarian cancer progression and chemopreventive potential of natural compounds [16,17,18,19,20,21,22,23,24,25,26,27]. The review is organized into sections covering etiology, risk factors and molecular mechanisms of ovarian cancer development. Then, cellular events and biochemical pathways targeted by natural compounds are discussed. Eventually, a focus on some relevant and widely studied molecules are presented.

## 2. Etiology of Ovarian Cancer

**Figure 1:**
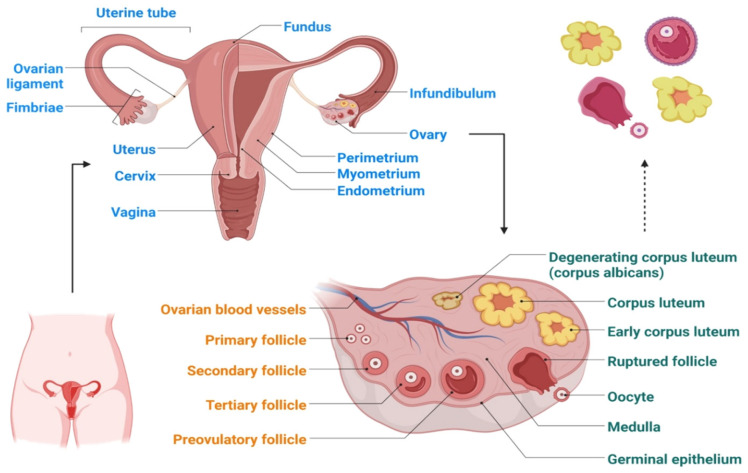
Schematic representation of the ovary structure.

Each ovary is approximately 3.5 cm long, 2 cm wide and 1 cm thick. It has an oval, solid structure that is about the size of an almond. The ovaries are located in ovarian fossae, which are small depressions on either side of the uterus in the lateral walls of the pelvic cavity. Characterizing the variation in the ovarian surface epithelium (OSE) in accordance with pre-cancer lesions (intraepithelial neoplasia) is quite difficult owing to the intra-abdominal localization of the ovaries (Figure 1) and prevalence of disease [28]. Therefore, knowledge regarding earlier genetic and molecular events that are linked with ovarian cancer is still very limited. As a result, the causes of ovarian cancer are not very clear, and this is particularly true for epithelial ovarian cancer. The alterations in the OSE are a contributing factor in ovarian cancer, as shown by the following evidence: (1) the OCP, a widely used strategy of ovarian cancer prevention, triggers cancer-preventive molecular pathways in the OSE [29]; (2) evidence of dysplastic, premalignant alterations in the OSE can be detected using conventional techniques [30]; (3) colocalization of dysplastic specimens in the OSE of ovaries with loss of cancer-suppressing action or overexpression of cyclooxygenase 2 [31]; and (4) evolution from a nonmalignant to a cancerous OSE in a some early ovarian malignancies [32].

Another hypothesis postulates that the cells that develop in the fallopian tube serve as the foundation for ovarian cancer growth [33]. This hypothesis, while it is still only a theory, is supported by the fact that the majority of ovarian cancer cells show histological features similar to those of the fallopian tube itself. Additionally, an abnormally high incidence of histologic and molecular markers linked to dysplasia at the fimbriated end of the fallopian tube is observed in preventive oophorectomy cases from high-risk women [34]. The probability of fallopian tube cancer development is noticeably increased in women who have a *BRCA*-related inherent ovarian disease risk. Furthermore, a thorough examination of the fallopian tubes in women with serous pelvic cancer has revealed a high likelihood of endosalpinx inclusion or concurrent tubal carcinomas. Similarly, p53 changes can be retrieved in both the pelvic and fallopian tube lesions, which suggests the possible hereditary character of the disease [35,36]. Additionally, p53 markers have been found in fallopian tubes removed for non-carcinogenic causes in women within a population bearing risk factors of ovarian cancer [37]. The fimbriated end of the fallopian chamber may evolve toward neoplasia when dysplastic cells shed from the OSE, or even when taking into consideration ovarian stromal materials provided during ovulation [38].

## 3. Risk Factors for Ovarian Cancer

Because of the rate of ovulatory cycles, it has been demonstrated that women who have early menarche (age < 12) and late menopause (age > 50) have a greater risk of ovarian cancer. In particular, early menarche and late menopause enhance the possibility of disease development by 1.1 to 1.5 times and 1.4–4.6 times, respectively. Breastfeeding, pregnancy, and the usage of ovulation-restricting oral contraceptives also all represent risk factors [39,40]. Endometriosis and ovarian cancer have been linked in epidemiological research, however the mechanism is uncertain [41]. Family history for ovarian cancer is known to be one of the most significant risk factors. *BRCA1* and *BRCA2* mutations have been associated with a high risk of ovarian and breast cancer development [39]. More specifically, women having mutations in *BRCA1* and *BRCA2* are thought to have elevated risk for the development of ovarian cancer [42]. Located on chromosome 17q21, *BRCA1* is an onco-suppressor gene, while *BRCA2* is located on chromosome 13q [39]. The codon is prematurely terminated when these genes are removed or inserted, resulting in a shortened protein. The mutation of such genes promotes uncontrolled cell proliferation because they play a role in chromatin remodeling. More specifically, *BRCA1* and *BRCA2* mutations have been linked to an increased risk of developing ovarian cancer by 50% and 20%, respectively [40,42,43,44,45,46,47,48,49,50,51]

## 4. Ovarian Cancer Carcinogenesis and Progression: Molecular Mechanisms

**Figure 2:**
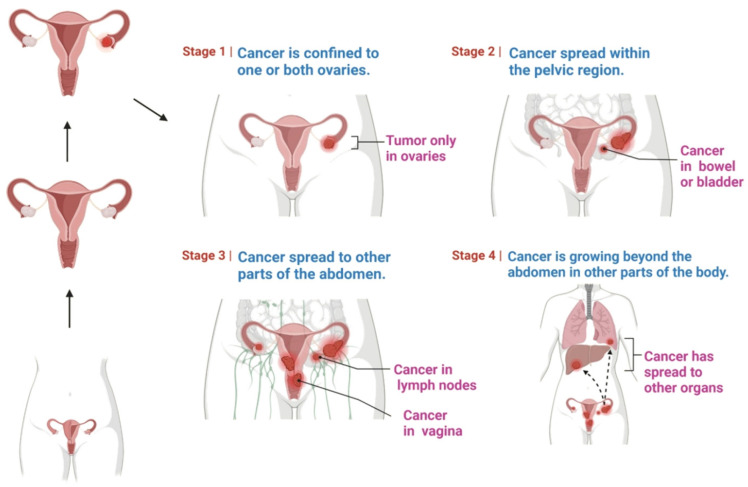
Stages of ovarian cancer, which vary from stage 1 to stage 4.

The stages of cancer progression are demonstrated in Figure 2: a higher number, such as stage 4, denotes that the cancer has progressed more widely. Several hypotheses have been formulated to explain the mechanisms underlying disease onset and progression.

In this context, the cancer stem cell hypothesis deserves particular attention as it has been introduced to explain many cancer complications, resulting in drug resistance, metastases, and recurrence, that are related to disease progression. Cancer stem cells are a small population of tumor cells that contribute to the formation of phenotypically diverse tumors. Their hallmarks have been recently demonstrated to be targeted also by natural compounds to combat cell invasion and recurrence [52,53,54].

Several other hypotheses have been proposed to clarify the mechanism of ovarian cancer progression. Repeated ovulation, when associated with repetitive ovarian epithelial damage and repair, raises the risk of DNA damage and carcinogenesis, according to the so-called ovulation theory [55]. As a result, having a higher number of ovulations raises the risk of ovarian cancer. In rats, hyper-ovulation raises the chance of ovarian cancer progression substantially. According to experimental studies, ovulation may produce carcinogenesis by stimulating multiple cellular actions [56]. Therefore, incessant ovulation increases the risk of mutagenicity due to transformation of injured OSE cells. Bradykinin and other vasoactive mediators, as well as leukocytes and prostaglandins, are stimulated during ovulation [57].

The gonadotrophin hypothesis suggests that increased levels of gonadotrophins drive epithelial neoplastic change either straightforwardly or by implication through steroidogenesis [58]. On the other hand, it has been noted that progestins can induce apoptotic pathways and decrease the chance of growth alterations, while estrogens can promote the development of the disease [59]. In this context, carcinogenesis may also likewise connected to the use of conceptive therapeutics [60].

Epithelial ovarian cancer cells can relocate to the peritoneal cavity, bringing about ascites and, potentially, immunosuppression that favors cancer development. Lysophosphatidic acid inhibits TNF receptor apoptosis-inducing ligand (TRAIL)-induced apoptosis by activating the Pl3K/Akt pathway [61]. In ascites and plasma samples from epithelial ovarian cancer specimens, a recent report found a huge change in cytokines, demonstrating in particular a unique variety in ascites cytokines [62]. Additionally, it has been noted that protein kinase C (PKC), Akt, lysophosphatidyl acid (LPA), and interleukin-6 (IL-6) are increased in ovarian cancer [63]. According to another report, ovarian cancer cells use the Akt/nuclear factor kappa B (NF-kB) pathway to produce IL-6, IL-8, and VEGF [64]. PKC likewise plays a critical role in the regulation of various pathways. More specifically, in ovarian cancer patients, PKC dysregulation has been related to carcinogenesis and resistance to treatments [65]. The abovementioned inflammatory agents and pathways result in increased ovarian cancer-related inflammation. Generally, inflammation then induces the production of various toxic oxidants that cause direct harm to DNA, proteins, and lipids, enhancing carcinogenesis [66]. In addition to this, inflammation is linked to increased cellular proliferation. Excessive cellular divisions then result in DNA repair replication errors, causing increasing mutagenesis [67]. Ovarian cancer cells release several inflammation mediators, including cytokines and interleukins [68]. In ovarian cancers, elevated levels of prostaglandins are observed as compared with normal cells [69], and prostaglandins promote cancer cell invasion at high concentrations [70]. Oxidative stress is another significant event associated with ovarian cancer. Compared with healthy women, patients with ovarian cancer show decreased antioxidant species and increased levels of oxidative stress [71]. More specifically, in ovarian cancer epithelial cells, some studies have reported oxidative stress conditions, with decreased concentrations of antioxidative enzymes. In addition to defective apoptosis, nitric oxide (NO), myeloperoxidase (MPO), NAD(P)H oxidase, and extended combinations of these enzymes have all been observed in ovarian cancer tissues. Besides, ovarian cancers have more elevated levels of caspase-3 nitrosylation, bringing about a huge decrease in caspase-3 capacity. MPO is a significantly relevant pro-oxidant chemical forming NO [72,73,74]. According to several experimental reports, high MPO levels have been detected in ovarian cancer cells [75]. At the molecular level, MPO directs apoptosis, severe reactions, and drug resistance [76]. MPO prompts the formation of reactive oxygen species (ROS), causing oxidative stress and influencing iron redox balance [72]. As a result, oxidative stress may play a crucial role in the progression of ovarian cancer [77].

## 5. Molecular Mechanisms Underlying Bioactivity of Natural Products

Mounting evidence demonstrates that plant-derived natural components like phytochemicals can have a role as adjuvants to conventional chemotherapy and may represent promising options for the future development of treatments against ovarian cancer [78,79].

The interest of researchers in the identification of small molecules acting as anticancer agents towards ovarian cancer is constantly growing, and this is testified by the increasing number of contributions in the field. In the 2019–2021 timeframe, some relevant reviews on this topic were published. Shafabakhsh and Asemi as well as Vafadar et al. reviewed the antiproliferative potential of one of the most widely studied natural compounds, quercetin, in the context of ovarian cancer [80,81]. On the other hand, Kubczak et al. reviewed the molecular targets for anticancer natural compounds identified so far, and organized their contribution into sections according to chemical classes [82]. Eventually, Wu et al. focused their attention on ovarian cancer, and classified the studied compounds according to the mechanisms by which natural molecules may act [83].

In this part of the review, we aim at providing a comprehensive and updated overview of natural compounds studied as anticancer agents in the context of ovarian cancer. With respect to previous contributions in the field, we expanded the list of possible molecular mechanisms according to the latest reports. Moreover, in the following sections, we provide a focus on the most promising compounds for which a higher amount of data can be retrieved in the recent literature. Eventually, we discuss the potential of semi-synthetic derivatives of natural compounds with enhanced anticancer activity.

More specifically, the current section briefly reports the role of natural constituents against ovarian cancer, and their proposed mode of action is also be discussed herein. In general, natural compounds potentially modulate chemotherapeutic resistance, autophagy, inflammation, propagation, and apoptosis [84]. A brief overview of the several cellular events is reported below. The studied molecules have been grouped according to the proposed mechanism of action.

### 5.1. Compounds Inducing Apoptosis and Cytotoxicity and Inhibiting Proliferation

Apoptosis is a kind of organized cell death, and it represents a crucial process for maintenance of homeostasis [85]. The induction of apoptosis and inhibition of cell proliferation are the main general mechanisms through which several natural compounds exert their anticancer role [86,87], and the main examples in the field of ovarian cancer are reported below.

Pro-apoptotic activity in ovarian cancer cell lines has been reported for procyanidins from cocoa [88], zeylenone from *Uvaria grandiflora* Roxb [89], and sanguiin H-6, a natural constituent present in red raspberry [90].

Similarly, methyl lucidone from *L. erythrocarpa* has cytotoxic effects and induced apoptosis in SKOV-3 and OVCAR-8 cell lines [91], while tanshinones from *Salvia miltiorrhiza* (Danshen), such as cryptotanshinone, tanshinone-I (Tan-I) and tanshinone-IIA (TII-A), were reported to induce apoptosis by interaction with TNF receptors. In particular, TII-A showed the highest activity [92].

Sulforafane (SFN) is a biologically relevant component found in cruciferous vegetables, including broccoli, and it suppressed cell growth by downregulating the cell cycle regulators cyclin D1 and cyclin-dependent kinases 4 and 6 [93].

Additionally, dihydroartemisinin (DHA), traditionally used to treat fever symptoms and recently investigated as a potential tool against severe acute respiratory syndrome-coronavirus 2 (SARS-CoV-2) [94,95], can be found in *Artemisia annua* [96] and induced apoptosis in ovarian cancer cells [97].

Reduced cell proliferation was also achieved with use of berbamine, an alkaloid obtained from *Berberis amurensis*, through the involvement of the Wnt/catenin signaling pathway [98].

Epigallocatechin gallate (EGCG), one of the main catechins found in green tea, inhibited the development and proliferation of OVCAR3 [99], as well as Pulchrin A from *Enicosanthellum pulchrum* [100].

Kadsuphilactone B, a nortriterpenoid from *Schisandra chinensis* (Turcz) B. [101], resveratrol [102] and curcumin [103], which will be discussed in a separate section of the review, represent other examples of compounds promoting ovarian cancer cell death.

Other naturally occurring mixtures, such as those containing silybin analogs, demonstrated a potential inhibitory effect on cancer development, including inhibition of elongation, pro-apoptotic effects, and cytotoxicity [104]. For a detailed list of extracts, the reader should refer to the review by Wu et al. [83].

Overall, the main involved mechanisms targeted by the abovementioned compounds include induction of DNA damage, caspase-3, reduction of Janus family tyrosine kinase (p-JAK), Akt phosporilation and SERCA, increased apoptosis-inducing factor (AIF), PARP and Bcl-2 family proteins.

### 5.2. Interference with Reactive Oxygen Species (ROS) Damage and with Nucleic Acid Repair

Excessive oxidative stress is generally believed to play a role in a wide range of diseases, from inflammation to cancer. Carcinogenesis has been connected to enhanced ROS formation and damage [105], and several studies have highlighted the involvement of antioxidant and radical scavenger properties of natural and synthetic compounds [106,107].

In various experiments, the abovementioned antioxidant SFN induced apoptosis in the OVCAR3, OVCAR4, OVCAR5, and SKOV3 cell lines and diminished cancer development in vivo [108].

Several flavones, including quercetin [109], and isoflavones have been previously reported to show antiproliferative activity [110]. Quercetin is a relevant natural compound that has been widely studied, and the properties of this molecule will be overviewed in another section of this review. It has been demonstrated that the isoflavone formononetin (FMN), which is found in red clovers and soy, has anticancer and cancer-preventive actions in a variety of cell types. FMN combats ROS and cell division [83,111].

DNA can be harmed directly or indirectly by events such as oxidative stress, radiations, alkylating agents, and a range of other chemotherapeutic techniques, but the capability of ovarian cancer cells to repair DNA damage is believed to be a crucial element in determining the resistance to chemotherapy [112].

In this context, sideroxylin from *Callistemon lanceolatus* induced apoptosis and reduced proliferation in ovarian cancer cells by influencing lipid peroxidation and ROS activity [113].

Additionally, berberine, another example of a common alkaloid that can be retrieved from several natural sources [114], which has been proven to stop cell division by interfering with DNA repair processes, inhibited the effects of PARP1, which is involved in oxidative states of damaged DNA [115].

Besides, alone or in combination with cisplatin, the abovementioned compound WFA induced the formation of reactive oxygen species (ROS) in A2780 ovarian cancer cells, which caused DNA harm. The compound acted in a synergistic cytotoxic manner with cisplatin, which formed DNA adducts [116].

On the other hand, in the context of the role played by nucleic acid sequences as targets for anticancer agents, aberrant RNAs have been discovered to play critical oncogenic roles in several human cancers. For example, astragalus polysaccharide (APS), a bioactive substance from *Astragalus membranaceus*, increased apoptosis while decreasing cell invasion targeting such sequences [117].

### 5.3. Modulation of Inflammation

It is now widely accepted that inflammation has a direct association with carcinogenesis as it contributes in initiation, proliferation, invasion, and metastasis [118]. Pro-inflammatory cytokines like TNF-α and IL-6 are blocked by anti-inflammatory compounds including baicalein, apigenin, curcumin, EGCG, genistein, luteolin, and wogonin [83]. Alongside, signal transducer and activator of transcription 3 (STAT-3) prevention, cyclooxygenase-2 (COX-2) inhibition, and nitric oxide synthase (iNOS) downregulation are considered as the main anti-inflammatory mechanisms of phytochemicals [119].

### 5.4. Suppression of Events Related to Disease Progression: Cell Migration and Angiogenesis

Cell migration and invasion are among the hallmarks of disease progression, and some natural compounds have been reported to target such events.

Among these, tetramethylpyrazine (TMP) from *Ligusticum wallichil* decreased cell viability and motility in SKOV-3 cells [120], and emodin, which is contained in several preparations of Chinese herbs, was found to suppress cell division, invasion, and migration by hindering the ILK/GSK-3β pathway [121].

Another event that contributes to disease progression is angiogenesis. BLP, the abovementioned mixture containing proanthocyanidins from Chinese bayberry leaves, is probably the most promising in this context, as it demonstrated an anti-angiogenic effect in the IOSE-364 ovarian cell line due to an inhibition of vascular endothelial growth factor (VEGF) [122].

Similarly, Tan-IIA, already mentioned above, interfered with disease progression in an A2780 xenograft model. Concerning the underlying mechanism of action, Tan-IIA promoted antiangiogenetic effects, mediated by the interference with VEGF, and induced apoptosis in the ID-8 and A2780 cell lines [123].

Several natural flavonoids were also reported to act on the EGF/VEGF pathway, including apigenin, taxifolin, luteolin, quercetin, genistein, kaempferol [124], harmine [125], and cranberry proanthocyanidin-1 [126].

### 5.5. Regulation of Tumor Micro Environment

The tumor microenvironment is a complex and dynamic combination of elements in which cancer cells are embedded. It comprises nonmalignant cells, the extracellular matrix and several cytokines, chemokines, and growth factors. Considering their multi-target action, natural compounds can modulate several aspects of the microenvironment. In particular, Dias et al. highlighted how natural derivatives can influence metabolic crosstalk to “re-educate” tumor microenvironment cells towards potential anticancer activity. In particular, curcumin, resveratrol, EGCG, shikonin, and phloretin were reported to alter the metabolism of stromal cells [127].

The abovementioned effect is achieved through the modulation of the expression of cancer-associated genes by the natural products, and this mechanism has also been reported to explain the anticancer activity of quercetin, berberine, and tanshinones [128].

In addition, β-escin was recently reported to combat ovarian cancer metastasis by targeting both cancer and stromal cells in the tumor microenvironment [129].

### 5.6. Other Mechanisms Related to Dysregulation of Cell Cycle

Dysregulation of the cell cycle is a relevant contributing factor in the carcinogenesis of ovarian cancer, and interference with the G0/G1 stages is the most commonly reported mechanism of natural compounds with an anticancer role targeting this process [130]. This mechanism was reported for asiatic acid from *Centella asiatica* [131], mentoflavone from *Selaginella tamariscina* [132], proanthocyanidins from Chinese bayberry leaves (BLPs) [133], and pulchrin A [134], which were found to combat cell proliferation and cancer progression, in particular by targeting such phases of the cell cycle.

Moreover, co-treatment with herbal extracts from *Fritillaria cirrhosa* (FC) and *Scutellaria baicalensis* (SB) resulted in G0/G1 stage cell cycle arrest also in OVCA 420 and OVCA 429 ovarian cancer cells [135].

Additionally, cucurbitacin-A, isolated from *Momordica charantia* L., was found to show anticancer potential, causing cell cycle arrest in the G2/M phase [136].

In this context, licorice plants contain large amounts of the flavonoid isoliquiritigenin (ISL). In OVCAR-5 and ES-2 cell lines, ISL also decreased cell proliferation in a dose- and time-dependent manner, targeting the G2/M phase of the cell cycle [137].

Autophagy is another physiological cell process that contributes to the maintenance of a normal cell cycle. According to increasing evidence, autophagy and ovarian cancer also appear to be connected [138]. Thus, natural compounds that help in modulating autophagy may find an application in ovarian cancer treatment. Among the natural constituents reported to act against ovarian cancer through this mechanism, *Emblica officinalis* (Amla) extracts [8], resveratrol [139], withaferin A (WFA) [140], and grifolin [141] were reported.

Moreover, Tan-I, a compound from the class of tanshinones, cited above, increased levels of the autophagy-related proteins beclin1, ATG7, and p62 as well as LC3II/LC3I and caspase-3 in A2780 and ID8, boosting apoptosis and inducing autophagy [142,143].

Finally, genistein promoted autophagy of caspase-independent cells [144] and induced apoptosis in cisplatin-sensitive and resistant ovarian cancer cells (A2780/CaOV3, ES-2).

### 5.7. Natural Constituents Modulating Resistance to Chemotherapeutic Agents

In ovarian cancer cells, plant-derived constituents were found to enhance sensitivity to chemotherapeutics, an aspect which, as anticipated, is crucial in this pathology.

For example, pre-treatment with either ellagic acid or resveratrol 48-h before cisplatin administration was reported to increase cytotoxicity of cisplatin itself in A2780CisR cisplatin-resistant cells, while synergistic treatment with either cisplatin–ellagic acid or cisplatin–resveratrol for 26 weekly cycles completely prevented cisplatin resistance in A2780 cells [145].

Moreover, in A2780 cells, SFN diminished the xenobiotic-reaction component (XRE). SFN also interferes with cell pH regulation and migration, and in this context it has been proposed as an agent to combat chemoresistance [146].

In doxorubicin-resistant human ovarian cancer cell lines (NCI/ADR-RES), treatment with RCM, also known as Korean dark raspberry, led to apoptosis through phosphorylation of c-Jun N-terminal kinase (JNK) [147]. In the same model, ellagic acid and quercetin, two phytochemicals also found in RCM as well as in many other natural sources, were shown to influence JNK and Akt phosphorylation, thus inducing apoptosis [148].

Finally, since therapy with WFA and doxorubicin reduced cell proliferation in xenograft mice models of ovarian cancer more effectively than WFA or doxorubicin alone, it has been postulated that WFA may be thought of as an adjuvant to standard doxorubicin therapy to minimize adverse effects [140].

**Figure 3:**
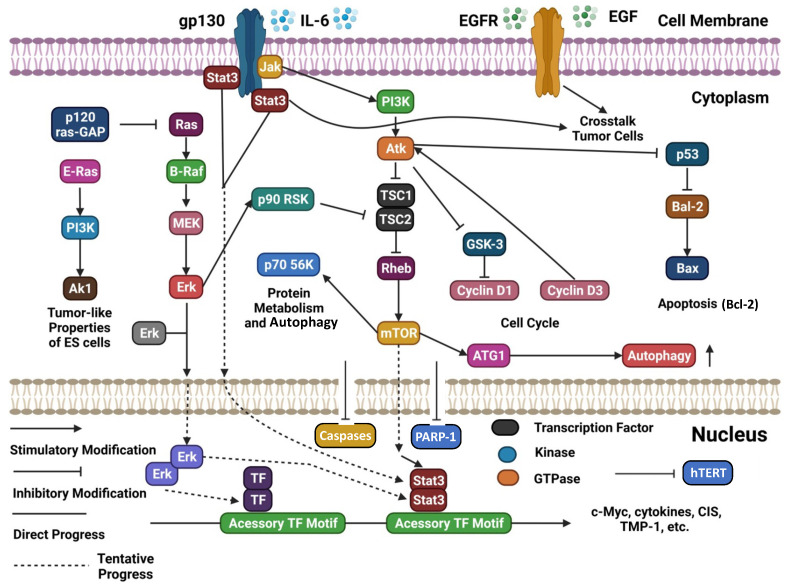
Schematic representation of several cellular signaling pathways potentially targeted by natural compounds (adapted and updated from [83]).

A schematic representation and summary of the pathways targeted by natural compounds and mentioned in this section are reported in Figure 3. Moreover, Table 1 summarizes the most recently reported updates on the molecular mechanisms underlying the activity of natural compounds and their derivatives in the context of ovarian cancer.

## 6. A Focus on Selected Natural Compounds with Promising Activity against Ovarian Cancer

**Figure 4:**
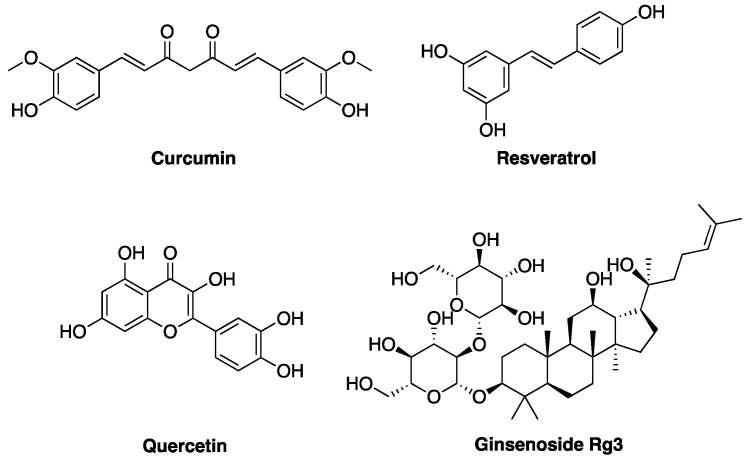
Chemical structures of curcumin, resveratrol, quercetin, and ginsenoside Rg3, the most promising natural compounds discussed in the review.

In the following part of the review, we focus on some of the most widely studied natural compounds, some of which have already been mentioned in previous paragraphs, with reported activity against ovarian cancer models (Figure 4). The compounds are presented to the reader according to a classification related to their chemical structure and natural origin. In particular, their potential as antiproliferative and anti-apoptotic agents, as well as the evidence concerning their anti-metastatic activity, are discussed. Eventually, a focus on semi-synthetic derivatives of natural compounds, designed to achieve improved anticancer activity, is presented.

### 6.1. Curcumin

The primary ingredient in South Asian and Indian curries is turmeric, which comes from the root of *Curcuma longa*. Turmeric has a long history of usage in India and China as a traditional medicine [155]. Curcumin and two others related curcuminoids, namely demethoxycurcumin and bisdemethoxycurcumin, are well-known and widely studied compounds contained in this plant. As indicated by several reports published throughout the years, curcumin and curcuminoids have strong anticancer effects due to the interaction with a combination of intracellular targets [156].

#### 6.1.1. Antiproliferative and Proapoptotic Activity

Excessive proliferation and unbalanced apoptosis are two signs of uncontrolled cell growth, and these events also occur in ovarian cancer, as discussed in the first part of this review. The protein kinase B/phosphatidylinositol 3-kinase (Akt/PI3K) signaling pathway is overactivated in ovarian cancer cells and supports cell proliferation and invasion [157].

In ovarian cancer cells, curcumin decreased Bcl-2 expression while it increased Bax and caspase-3, causing cell cycle arrest in the G2/M stage and consequent cell death [158]. According to Watson et al., curcumin activates caspase-8 and caspase-9 first, and then caspase-3 to exert this activity [159]. Moreover, the authors found that curcumin decreased Akt phosphorylation, Bcl-2, and survivin, an anti-apoptotic protein. When phosphorylated, STAT-3 advanced malignant growth by promoting cell proliferation and hindering apoptosis [160]. According to Saydmohammed et al., curcumin also reduced STAT-3 phosphorylation, which regulates the growth of ovarian cancer cells [161]. Curcumin regulated STAT-3 phosphorylation and enhanced interleukin (IL)-6 and IL-8 release, which decreased ovarian cell motility [162]. Seo et al. reported that curcumin influenced Ca^2+^ homeostasis in ovarian cancer cells [103]. Curcumin also interferes with miRNAs, short non-coding RNA sequences regulating target genes post-transcriptionally [163]. According to Du et al., treatment with dimethoxy-curcumin sustained the levels of miR-551a, inducing apoptosis in ovarian cancer cells [164]. Additionally, when dihydroartemisinin and curcumin were combined, miR-124 was upregulated and its target, midkine, which promotes carcinogenesis and is overexpressed in infections, was downregulated [165], causing cell cycle arrest and apoptosis. Additionally, curcumin increased apoptosis and stopped the growth of ovarian cancer cells by targeting miR-9 [166].

Thus, as can be deduced from the results of the high number of reports concerning curcumin published in this context, it can be postulated that the compound may act through a combination of mechanisms at the molecular level to exert its antiproliferative activity.

#### 6.1.2. Anti-Metastatic Activity

Unregulated cancer cells, also in the case of ovarian cancer, can spread to different organs [4], and in this context matrix metalloproteinases (MMPs), a type of proteolytic protein, promote the development of ovarian cancer [167]. By reducing the phosphorylation of FAK, MMP-9, and Rab coupling protein, curcumin inhibited SKOV3 cell invasion [168]. Moreover, bisdemethoxycurcumin reduced metastasis-related proteins such as MMP-2, MMP-9, and vascular cell bond particle 1 (VCAM-1) in SKOV3 cells by controlling oxidative stress and inactivating the NF-κB pathway [169]. The cooperation of VCAM-1 and integrin has been shown to play a role in ovarian cancer cell intrusion and metastasis [170].

### 6.2. Resveratrol

Resveratrol is a polyphenolic compound that can be found in grapes, peanuts, and plants such as *Polygonum cuspidatum* [171]. Resveratrol improves heart disease and conditions affecting the nervous system and kidneys, and it is reported to have several other beneficial properties [172,173]. This compound has been widely studied throughout the years for its biological roles, and it has been cited previously in the current review. In this section, a brief overview of the reports concerning its antiproliferative and anti-metastatic activities are reported.

#### 6.2.1. Antiproliferative and Proapoptotic Activity

In a mouse model, resveratrol diminished glucose uptake by cancer cells [174]. Resveratrol influenced GSK3β in ovarian cancer cells, reducing protein glycosylation [175]. GSK3β phosphorylated and deactivated glycogen synthase, thus regulating glucose storage [176]. Tino et al. [177] found that the combined use of resveratrol and acetyl resveratrol efficiently retarded the growth of ovarian cancer cells, and that this effect was accomplished by decreased NF-κB protein [177]. Besides GSK3, in ovarian cancer cells, resveratrol reduced the phosphorylation of Akt, and increased the extracellular signal-coordinating kinase (ERK) [102].

#### 6.2.2. Anti-Metastatic Activity

By reducing integrin levels, resveratrol has been shown to hinder the ability of ovarian cancer cells to invade the peritoneal mesothelium, thus preventing metastasis [178]. Resveratrol may limit the interaction between ovarian cancer cells and mesothelial cells by preventing the motility of the firsts, and, in particular, downregulation of VEGF in hypoxic conditions appears to be involved [179]. In fact, increased VEGF production has been shown to be related to metastasis in ovarian cancer cells [180].

### 6.3. Ginsenosides

The major pharmacologically active components of ginseng, ginsenosides, have antioxidant and anticancer properties [173,181]. Ginsenoside Rg3 and Rb1 especially have been reported to display anticancer activity [182].

#### 6.3.1. Antiproliferative and Proapoptotic Activity

According to Li et al., ginsenoside Rg3 decreased ovarian disease cell glycolysis by downregulating phospho-STAT-3 [183]. Additionally, the compound triggered the upregulation of miR-603 in ovarian cancer cells by inhibiting DNA methylation. The same natural molecule also influenced hexokinase-2 activity [184].

However, the use of ginsenoside Rg3 is a rather debated issue, as recent reports showed that the use of low concentrations of the compound stimulated cell proliferation, while high concentrations were needed to achieve anticancer effects [185].

#### 6.3.2. Anti-Metastatic Activity

HIF-1α is a dimeric protein that plays a role in hypoxic conditions and metastasis [186]. Epithelial–mesenchymal transition (EMT), which is related to cell–cell adhesion, frequently takes place prior to the onset of ovarian cancer. Liu et al. observed that ginsenoside 20(S)-Rg3 counteracted EMT and downregulated HIF-1 through interference with the ubiquitin–proteasome pathway [183]. Additionally, it was discovered that ginsenoside Rg3 increased prolyl hydroxylase protein 1 and resulted in HIF-1α degradation [187]. Moreover, a reduction in cell intrusion capability was observed upon treatment with this compound [184].

Furthermore, ginsenoside Rb1 reduced hypoxia-induced EMT in ovarian cancer cells by downregulating miR-25. More specifically, it prevented the production of EP300, a transcriptional activator of E-cadherin, a crucial molecule for epithelial cell attachment, from being suppressed by miR-25, which would have had an anti-metastatic impact [188]. By interaction with actin microfilaments in the cytoplasm through α- and β-catenin, E-cadherin is associated with the adhesion of epithelial cells [189].

### 6.4. Quercetin

**Figure 5:**
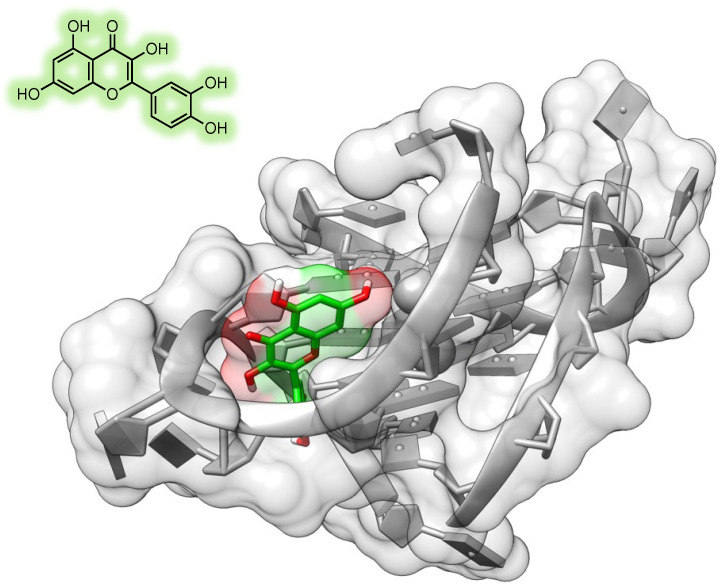
Quercetin structure and predicted binging mode to a G-quadruplex DNA sequence (Protein Data Bank ID: 3CE5; adapted from [190]). The artwork was produced using UCSF Chimera [191].

Quercetin is one of the most widely studied naturally occurring flavonoids, which is known to possess a plethora of biological properties through several mechanisms including interaction with DNA (Figure 5) and several protein targets [190], and this section of the review is focused on the reports on anticancer activity of this compound in ovarian cancer models. Shafabakhsh and Asemi [93] and by Vafadar et al. [94] recently reviewed the anticancer properties of quercetin, and the reader is invited to refer to these reviews for a more comprehensive overview of the molecular mechanisms of this compound.

Liu et al. examined the effects of quercetin on apoptosis in an ovarian cancer mice xenograft model and demonstrated that quercetin caused mitochondrial apoptosis [192]. Furthermore, by causing endoplasmic reticulum (ER) stress, quercetin triggered mitochondria-mediated apoptosis in ovarian cancer cells. Quercetin affected ER stress, apoptosis, and autophagy via the p-STAT3/Bcl-2 center. In general, quercetin downregulates the growth of metastatic ovarian cancer cells through the induction of apoptotic conditions. In particular, the flavonoid increases the activity of apoptotic species including caspase-3, caspase-9, and cytochrome c. Moreover, pro-apoptotic proteins Bid, Bax, and Bad are also involved. Concerning this biomolecular mechanism, Bad and Bid promote the oligomerization of Bax and of the protein Bak, and this event triggers the permeabilization of the outer mitochondrial wall. Bid can directly trigger apoptosis, while Bad interacts with anti-apoptotic Bcl-2 proteins, thus lowering the threshold for induction of apoptosis. As a result, their equilibrium has an effect on the neoplastic shift in the human endometrium [193]. Like curcumin, quercetin causes mitochondrial-mediated apoptosis and thus limits the proliferation of metastatic ovarian cancer cells [194]. Moreover, our group recently demonstrated that quercetin, as well as its glycoside rutin, can target specific DNA sequences and arrangements in vitro, demonstrating that the compound could influence gene expression [190].

As for natural compounds and drugs in general, the bioavailability and formulation/delivery system may be crucial for obtaining the biological effects. In this context, another study investigated the antiproliferative potential of a quercetin-based nano-formulation. Both in vitro and in mice xenograft models, this specific form of quercetin reduced the development of ovarian cancer cells. Furthermore, it has been noted that quercetin from the nano-formulation activated caspase-3, caspase-9, and Bax while inhibiting MCL-1 and Bcl-2 to enhance apoptosis [195].

Additionally, several studies were aimed at understanding the synergistic benefits of quercetin when used in combination with various chemotherapeutics. In an in vitro/in vivo investigation, Gong et al. [196] examined the effects of quercetin combined with radiation on ovarian cancer. Exposure of quercetin made ovarian cancer cells undergo ER stress, and there was also an increase in p53, p21, and Bax expression, a reduction of Bcl-2 expression, and an increase in DNA damage.

Quercetin coupled with radiation dramatically decreased the growth of cancer cells and activated p53 in a xenograft ovarian cancer model. In another study, pretreatment with quercetin sustained the cytotoxic activity of cisplatin in ovarian cancer patients. In particular, quercetin increased ER stress, decreased STAT3 phosphorylation, and decreased Bcl-2 expression. Quercetin supported the anticancer impacts of cisplatin also in a xenograft mice model, suggesting a potential role for quercetin as a promising adjuvant medication for ovarian cancer treatment [197].

Other formulations of quercetin, such as PEGylated liposomal quercetin (lipo-quercetin), were tested in vitro and in vivo in models of both cisplatin-sensitive and cisplatin-resistant human ovarian cancer. Studies conducted in vitro revealed that the presence of lipo-quercetin caused cell cycle arrest and apoptosis in both kinds of cancer cells. Moreover, lipo-quercetin was more effective than free quercetin in mice xenograft models [198]. Several studies examined the impact of quercetin on cell cycle progression [199], and it was reported that the compound regulated 1-phosphatidylinositol 4-kinase (PI kinase) activity and lowered inositol-1,4,5-triphosphate (IP3) levels, thus confirming its effect on the cell cycle [200]. Moreover, quercetin was recently studied as an anti-metastatic agent in the context of ovarian cancer [201].

In the context of quercetin derivatives, an in vitro study examined the effects of 3,4′,7-O-trimethylquercetin (34′7TMQ) on the growth and progression of ovarian cancer cells, and the compound diminished ovarian cancer cell invasion [202].

Importantly, recent studies showed that quercetin can help in mitigating the side effects of chemotherapeutic agents including cisplatin, 5-fluorouracil, taxol, and pirarubicin. While other, less recent, research reports showed quercetin to be directly effective in the treatment of ovarian cancer, especially when paired with other drugs; it has been later demonstrated that low doses of quercetin increase antioxidant enzymes and reduce ROS-mediated anti-neoplastic drug toxicity [203]. In this context, another in vitro study in ovarian cancer cells supported the synergistic effect of quercetin when administered in combination with cisplatin [204].

**Figure 6:**
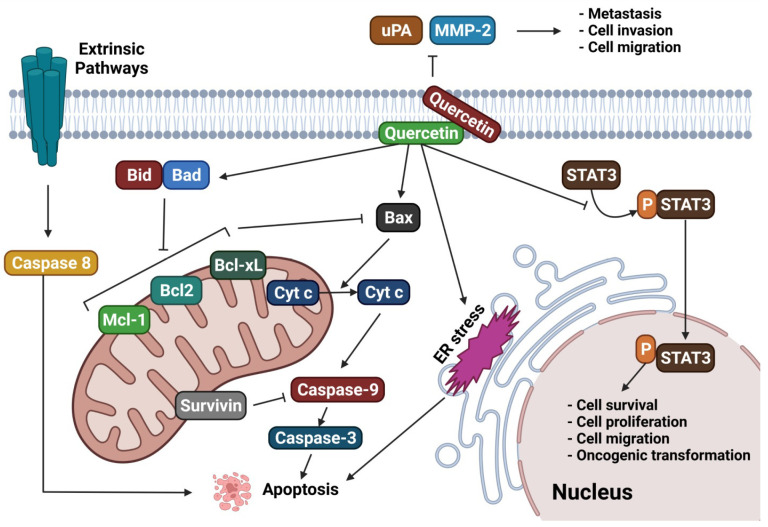
Quercetin targets several signaling pathways within the cells, representing a potential therapeutic agent against ovarian cancer (adapted from [80]).

The findings reported in this section suggest that quercetin and its formulations have anticancer potential against ovarian cancer through a combination of several mechanisms, which are outlined in Figure 6. Table 2 reports an update on findings concerning the potential of quercetin as an anticancer agent, in particular in the context of ovarian cancer. As can be noted from the records reported in the table, great efforts are currently focused on improving drug-likeness features and delivery strategies for this compound, together with the investigation of synergistic and potentiation effects with respect to traditional anticancer agents.

### 6.5. Semi-Synthetic Compounds

As anticipated, natural compounds are endowed with unique features in terms of chemical diversity and are often characterized, as highlighted by the overview presented in the previous sections of this review, by the capability of targeting a combination of biochemical pathways within the cell. Nevertheless, such compounds may also be characterized by poor bioavailability, lack of drug-likeness features, limited availability from natural sources, and poor specificity. Thus, research has recently focused on the design of semi-synthetic or synthetic derivatives of natural compounds with improved performances to address these issues [208].

Several examples of semi-synthetic investigational anticancer agents are presented in the literature. Napabucasin and other derivatives of naphthoquinones from *Handroanthus impetiginosus* were tested against cancer cells. The compounds inhibited STAT3, induced apoptosis, and stimulated ROS production [209]. Another example is represented by the paper from Nadysev et al. that reported the synthesis and characterized the biological activity profile of 4-aminomethyl derivatives of heliomycin, a metabolite from *Actinomyces flavochromogenes* var. *heliomycini* and *Streptomyces resistomycificus*. The molecules were tested against a set of cell lines and showed improved water solubility and antiproliferative efficacy with respect to the natural compound [210].

More specifically, concerning therapeutic approaches against ovarian cancer, Li et al. very recently reported a set of derivatives of celastrol, a compound isolated from *Tripterygium* species. This molecule has promising anticancer properties, but it is endowed with suboptimal pharmacological properties due to poor water stability, low bioavailability, and toxicity. The authors modified the structure to obtain drug-like compounds targeting the STAT3 pathway and showing anti-proliferative activity through induction of apoptosis and reduction of cell migration [152].

Previously, Chen et al. studied the synthetic compound FBA-TPQ, a derivative of the marine pyrroloiminoquinone alkaloid makaluvamine, which is isolated from sponges of the genera *Zyzzya*. FBA-TPQ exhibited anticancer activity against OVCAR-3 ovarian cancer cells through ROS species, p53-MDM2, and PI3K-Akt pathways. Moreover, minimal toxicity was observed in non-tumorigenic human IOSE-144 cells, and in vitro data were supported by in vivo studies in xenograft models [151].

Verticillins are another class of fungal metabolites, and several ester derivatives of verticillin H were prepared and tested against a panel of cancer cell lines, including OVCAR-3. The compounds showed cytotoxic activity in the nanomolar range [154].

Cycleanine is a bisbenzylisoquinoline macrocyclic alkaloid from *Triclisia subcordata*. Uche et al. synthesized a small pool of aminoalkyl derivatives that were tested against ovarian cancer cells. The molecules showed anticancer activity through activation of caspases 3/7 and cleavage of PARP [149].

**Figure 7:**
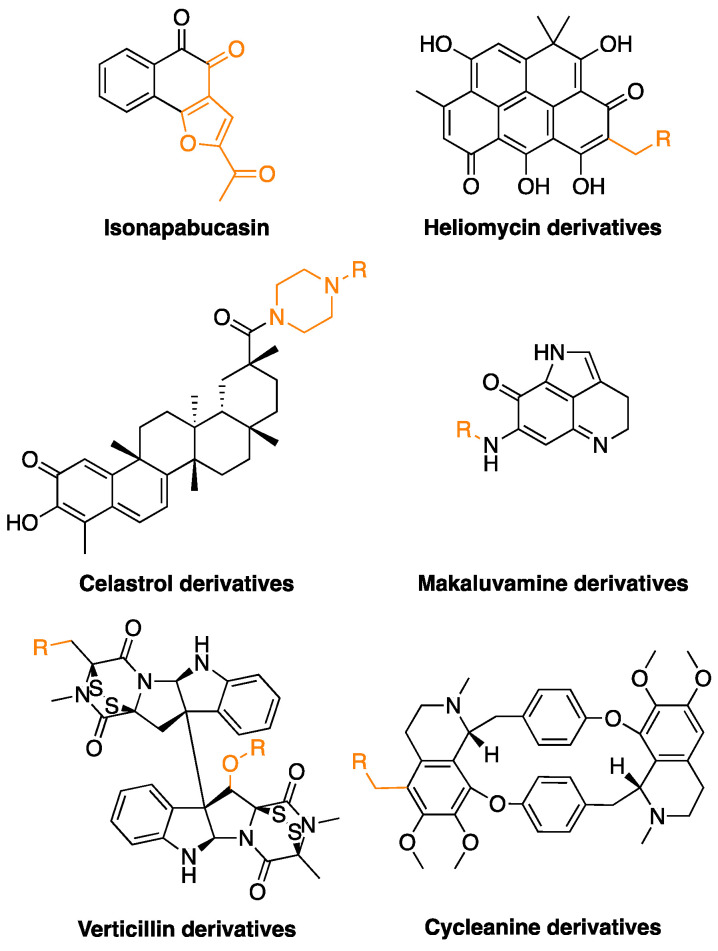
Chemical structures of the discussed semi-synthetic derivatives of natural compounds. The parts of the molecules that have been modified are highlighted in orange.

The chemical structures of the most promising semi-synthetic compounds are shown in Figure 7, and the main findings in this context are outlined in Table 1.

## 7. The Point of View of the Medicinal Chemist

Although it is widely established that first-line conventional chemotherapy has a therapeutic effect in many ovarian cancer patients, drug resistance typically limits the efficacy of treatment regimens. Natural products have been investigated in ovarian cancer models both per se and in adjuvant treatment, with positive outcomes in several cases, as overviewed in the previous paragraphs. As laid out in the reported studies, such compounds, belonging to several different chemical classes and acting through a combination of mechanisms, may effectively hinder cancer progression according to in vitro and in vivo studies. Importantly, moreover, natural compounds alone or in combination, boost chemotherapy efficacy while lowering toxic and side effects, potentially allowing a more promising outcome.

Three major observations can be drawn from this literature overview, considering the topic from the point of view of the medicinal chemist. First, the multi-target mechanism of natural compounds represents a valuable resource, but the lack of selectivity may represent a drawback for the development of novel therapeutic approaches. Second, it must be noted how many natural compounds, besides showing anticancer activity per se, also possess potentiating/synergistic properties with respect to other drugs. Additionally, they can help in re-sensitizing resistant cancer cells. Eventually, it must be considered that natural compounds should represent the starting point for compound optimization, as testified by the growing interest towards semi-synthetic derivatives with enhanced drug-likeness and performances. In fact, as overviewed in Section 6 of this review, the development of semi-synthetic compounds is pushing the efficacy of nature-derived molecules in the nanomolar range, even if every case is peculiar. Moreover, the efforts of medicinal chemists, besides improving antiproliferative activity, are pointed towards improving water solubility, stability, bioavailability, and toxicity profiles.

However, a major pitfall is still present and must not be ruled out, as very limited data support the clinical use of the natural compounds discussed in this paper. In fact, few clinical studies have assessed the anticancer effects of natural molecules, even for the most studied ones such as quercetin, particularly in the field of ovarian cancer [81,211]. In other cases, compounds that have been demonstrated to be effective in in vivo models of ovarian cancer were tested in clinical trials, but with different anticancer indications. This is, for example, the case for β-escin [212]. Nevertheless, there are also compounds that have been more widely studied in clinical trials such as resveratrol, which, anyway, is affected by limitations in terms of bioavailability [213].

## 8. Conclusions and Future Perspectives

This review overviewed and summarized the anticancer potential of plant-derived molecules in ovarian cancer models. The compounds discussed in this paper bear a variety of different chemical scaffolds, as can be expected for molecules of natural origin. In this connection, the main classes comprise phenolic components, flavonoids, steroid glycosides, terpenoids, chalcones, and several alkaloids.

Similarly, the underlying molecular mechanisms for the different compounds are very diverse, and they include autophagy and apoptosis induction, ROS activity, inhibition of cell invasion, angiogenesis, and metastasis [214]. Most importantly, natural molecules often act through a combination of the abovementioned mechanisms.

Nevertheless, more exploration is required to estimate and understand the effective potential of natural substances in pre-clinical and clinical trials. In particular, some major points must be addressed, including (i) assessment of dose for use of natural compounds in ovarian cancer; (ii) usage of natural compounds as co-treatments with chemotherapy, radiotherapy, and other immunotherapies [215]; (iii) use of a combination of natural compounds acting through different and ideally synergistic mechanisms; and (iv) advanced formulation studies to improve bioavailability of the molecules, thus paving the way for the potential application of natural and nature-inspired compounds as antiproliferative agents against ovarian cancer.

## Figures and Tables

**Table 1:** Overview of natural substances showing anticancer properties against ovarian cancer models. This table is intended as an addendum to the one reported by Wu et al. [83], thus updated records were included. The reader is invited to refer to the abovementioned review for a more comprehensive overview with the corresponding references. The table also includes semi-synthetic derivatives of natural compounds that showed antiproliferative activity and that are discussed in Section 6.5 of the current review.

Compound	Source	Chemical Structure of the Representative Component	Classification	Model	Mechanism of Action	References
Aminoalkyl derivatives of cycleanine	Triclisia subcordata	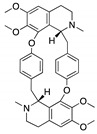 (Cycleanine)	Bisbenzylisoquinoline macrocyclic alkaloid	Cell lines	activation of caspases 3/7, cleavage of PARP	[149]
Berberine	European barberry, goldenseal, goldthread, Oregon grape, phellodendron, and tree turmeric	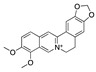	Alkaloid	A2780, HEY, HO8910	Triggering oxidative DNA damage, targeting of cancer stem cells	[52,115]
Epigallocatechin gallate (EGCG)	Green tea	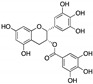	Flavonoid	SKOV3-ip1, SKOV3TR-ip2	Reduction of hTERT and Bcl-2, alteration of the metabolism of stromal cells	[99,127,150]
FBA-TPQ (derivative of makaluvamines)	*Zyzzya* sponges	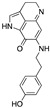 (Makaluvamine scaffold)	Pyrroloiminoquinone alkaloid	in vitro and in vivo (xenograft)	ROS species, p53-MDM2 and PI3K-Akt pathways	[151]
Phloretin	Apple tree leaves	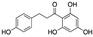	Dihydrochalcone	in vitro	Alteration of the metabolism of stromal cells	[127]
Semi-synthetic derivatives of celastrol	*Tripterygium* species	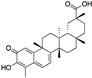 (Celastrol)	Nortriterpen quinone	in vitro	STAT-3 pathway, induction of apoptosis, reduction of cell migration	[152]
Shikonin	*Alkanna tinctoria*	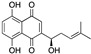	Naphthoquinone	A278 cells, in vitro	Alteration of the metabolism of stromal cells	[127,153]
Tanshinones	*Salvia miltiorrhiza*	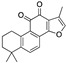 (Tanshinone IIA)	Terpenoid/Abietane	A-549, TOV-21G	Growth capacity is inhibited by reducing cell viability, alteration of the microenvironment	[92,123,128,143]
Verticillin H esters	Fungi	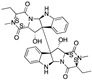	Verticillins	OVCAR-3	Reduced cell proliferation	[154]
β-escin	horse chestnut seed	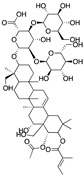	Pentacyclic triterpenoid saponin	in vitro and in vivo	Alteration of the microenvironment	[129]

**Table 2:** Update on experimental data reported in the literature for the activity of quercetin in the ovarian cancer model. This table is intended as an addendum to the ones reported by Shafabakhsh and Asemi [80] and by Vafadar et al. [81], thus updated records were included. The reader is invited to refer to the abovementioned reviews for a more comprehensive overview, with the corresponding references.

Compound/Formulation	Ovarian Cancer Model	Type of Study	Major Findings and Mechanisms	Reference
Graphene oxide polyvinylpyrrolidone-quercetin-gefitinib (GO-PVP-QSR-GEF)	Ovarian cancer cells	In vitro	Synergistic cytotoxic effect	[205]
Micellar(nanostructures) resveratrol (R):quercetin (Q) (mRQ)	Xenograft model	In vivo	Improvement of the efficacy of adriamycin	[206]
Quercetin	-	In vitro	Human telomeric G-quadruplex stabilization	[190]
Quercetin	Ovarian cancer cells	In vitro	Attenuation of metastatic ability	[201]
Quercetin micelle and thermosensitive hydrogel drug delivery system	SKOV-3 cells and animal model	In vitro and in vivo	Enhanced cytotoxicity	[207]

## Data Availability

Data sharing not applicable. No new data were created or analyzed in this study.
